# (1*S*,2*R*,3*S*,6*S*,7*R*)-3,7,11,11-Tetra­methyl-6,7-epoxybi­cyclo­[5.4.0]undecane-2-ol

**DOI:** 10.1107/S1600536814008642

**Published:** 2014-04-26

**Authors:** Mohamed Loubidi, Ahmed Benharref, Lahcen El Ammari, Mohamed Saadi, Moha Berraho

**Affiliations:** a Laboratoire de Chimie des Substances Naturelles, "Unité Associé au CNRST (URAC16)", Faculté des Sciences Semlalia, BP 2390 Bd My Abdellah, 40000 Marrakech, Morocco; b Laboratoire de Chimie du Solide Appliquée, Faculté des Sciences, Université Mohammed V-Agdal, BP 1014, Avenue Ibn Battouta, Rabat, Morocco

## Abstract

The title compound, C_15_H_26_O_2_, was synthesized from β-himachalene (3,5,5,9-tetra­methyl-2,4a,5,6,7,8-hexa­hydro-1*H*-benzo­cyclo­heptene), which was isolated from the Atlas cedar (*cedrus atlantica*). The mol­ecule is built up from a seven-membered ring to which a six- and a three-membered ring are fused. The seven- and six-membered rings each have a twist-boat conformation. In the crystal, O—H⋯O hydrogen bonds link the mol­ecules into zigzag chains running along the *b-*axis direction.

## Related literature   

For background to β-himachalene, see: El Haib *et al.*;(2011 ▶). For the reactivity of this sesquiterpene and its derivatives, see: El Jamili *et al.* (2002 ▶); Benharref *et al.* (2013 ▶); Ourhriss *et al.* (2013 ▶). For their potential anti­fungal activity against the phytopathogen *Botrytis cinerea*, see: Daoubi *et al.* (2004 ▶). For puckering parameters, see: Cremer & Pople (1975 ▶).
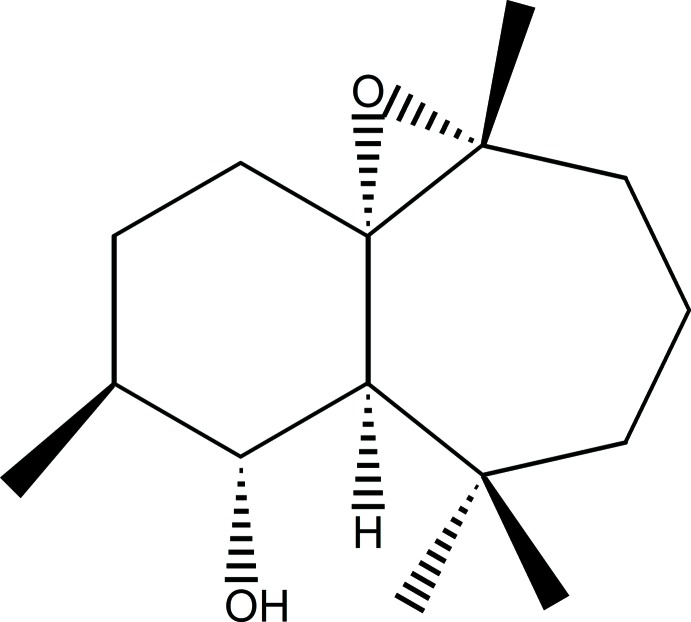


## Experimental   

### 

#### Crystal data  

C_15_H_26_O_2_
*M* *_r_* = 238.36
Monoclinic, 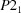

*a* = 5.9617 (10) Å
*b* = 12.068 (2) Å
*c* = 9.5909 (15) Å
β = 95.789 (8)°
*V* = 686.48 (19) Å^3^
*Z* = 2
Mo *K*α radiation
μ = 0.07 mm^−1^
*T* = 298 K
0.38 × 0.11 × 0.10 mm

#### Data collection  

Bruker X8 APEX diffractometer
11541 measured reflections
3036 independent reflections
2698 reflections with *I* > 2σ(*I*)
*R*_int_ = 0.031

#### Refinement  

*R*[*F* ^2^ > 2σ(*F* ^2^)] = 0.045
*wR*(*F* ^2^) = 0.131
*S* = 1.06
3036 reflections
162 parameters
1 restraint
H atoms treated by a mixture of independent and constrained refinement
Δρ_max_ = 0.34 e Å^−3^
Δρ_min_ = −0.16 e Å^−3^

### 

Data collection: *APEX2* (Bruker, 2009 ▶); cell refinement: *SAINT* (Bruker, 2009 ▶); data reduction: *SAINT*; program(s) used to solve structure: *SHELXS97* (Sheldrick, 2008 ▶); program(s) used to refine structure: *SHELXL97* (Sheldrick, 2008 ▶); molecular graphics: *ORTEP-3 for Windows* (Farrugia, 2012 ▶); software used to prepare material for publication: *PLATON* (Spek, 2009 ▶) and *publCIF* (Westrip, 2010 ▶).

## Supplementary Material

Crystal structure: contains datablock(s) I. DOI: 10.1107/S1600536814008642/bt6976sup1.cif

Structure factors: contains datablock(s) I. DOI: 10.1107/S1600536814008642/bt6976Isup2.hkl

CCDC reference: 997713

Additional supporting information:  crystallographic information; 3D view; checkCIF report

## Figures and Tables

**Table 1:** Hydrogen-bond geometry (Å, °)

*D*—H⋯*A*	*D*—H	H⋯*A*	*D*⋯*A*	*D*—H⋯*A*
O1—H11⋯O2^i^	0.72 (3)	2.11 (3)	2.820 (2)	171 (3)

Symmetry code: (i) 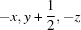.

**Fig. 1.**
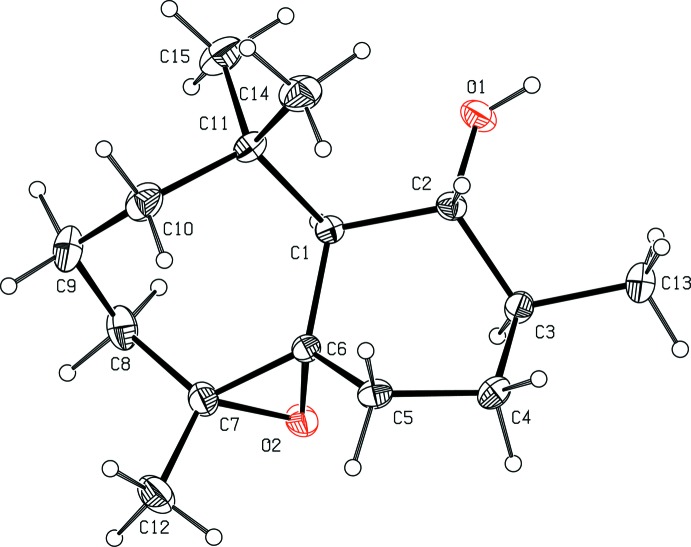
: Molecular structure of the title compound with the atom-labelling scheme. Displacement ellipsoids are drawn at the 30% probability. level. H atoms are represented as small spheres of arbitrary radii.

**Fig. 2.**
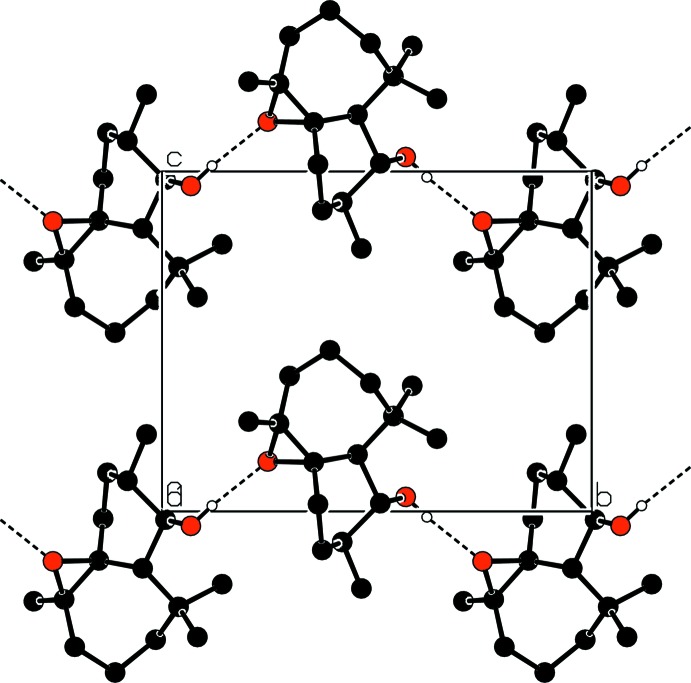
: Packing view showing the O–H···O hydrogen bonds as dashed lines. H atoms not involved in hydrogen bonding have been omitted for clarity.

## supplementary crystallographic information

### 1. Comment

This work is a part of our ongoing program concerning the valorization of the most abundant essential oils in Morocco, such as Cedrus atlantica. This oil is made up mainly (75%) of bicyclic sesquiterpene hydrocarbons, among which is found the compound β-himachalene (El Haib *et al.*, 2011). The reactivity of this sesquiterpene and its derivatives has been studied extensively by our team in order to prepare new products having biological properties (El Jamili *et al.*, 2002; Benharref *et al.*, 2013; Ourhriss *et al.*, 2013). Indeed, these compounds were tested, using the food poisoning technique, for their potential antifungal activity against phytopathogen Botrytis cinerea (Daoubi *et al.*, 2004). The molecule of the title compound (Fig.1) is built up from two fused six- and seven-membered rings, both rings have a twist-boat conformation as indicated by the total puckering amplitude QT = 0.7821 (19) Å and spherical polar angle θ = 92.53 (15)° with φ = 272.91 (14)°, for the six-membered ring, and QT = 1.1479 (21), θ = 87.76 (10), φ2 = -148.25 (21), φ3 = 27.36 (3) for the seven-membered ring (Cremer & Pople, 1975). In the crystal, O—H···O hydrogen bonds links the molecules into zigzag chains running along the *b* axis (Table 1, Fig. 1).

### 2. Experimental

Diborane is prepared by addition at 0 °C of 2.5 g (17 mmol) of boron trifluoride etherate in 0.5 g (12.6 mmol) of sodium borohydride in 30 ml of diglyme. Diborane formed is driven by a stream of dry nitrogen in 2 g (10 mmol) of β-himachalene dissolved in 20 ml of tetrahydrofuran at 273K. This takes about 4 h. 2 ml of sodium hydroxide 3 N is then added carefully between 263K and 273K in 15 minutes and then 2 ml of 30% hydrogen peroxide in the vicinity of 298K. The reaction mixture was then extracted with diethyl ether, the organic phase is washed to neutrality and the solvent was evaporated under vacuum. The residue obtained is chromatographed on a column of silica gel with hexane-ethyl acetate (95:5), which allowed the isolation of pure (1*S*, 2*R*, 3S, 6S, 7*R*)-6,7-epoxy-3,7,11,11- tetramethylbicyclo[5.4.0] undecane-2-ol with a yield of 25% (600 mg 2.5 mmol). The title compound was recrystallized from its cyclohexane solution.

### 3. Refinement

Except H11, which was freely refined, all H atoms were fixed geometrically and treated as riding with C—H = 0.96 Å (methyl),0.97 Å (methylene), 0.98 Å (methine) with *U*_iso_(H) = 1.2*U*_eq_(methylene, methine) or *U*_iso_(H) = 1.5*U*_eq_(methyl). The methyl groups were allowed to rotate, but not to tip. In the absence of significant anomalous scattering, the absolute configuration could not be reliably determined and any references to the Flack parameter were removed. The absolute configuration of the chiral centres was arbitrarily set.

### Crystal data

**Table:**

C_15_H_26_O_2_	*F*(000) = 264
*M**_r_* = 238.36	*D*_x_ = 1.153 Mg m^−^^3^
Monoclinic, *P*2_1_	Mo *K*α radiation, λ = 0.71073 Å
Hall symbol: P 2yb	Cell parameters from 3036 reflections
*a* = 5.9617 (10) Å	θ = 2.7–27.1°
*b* = 12.068 (2) Å	µ = 0.07 mm^−^^1^
*c* = 9.5909 (15) Å	*T* = 298 K
β = 95.789 (8)°	Needle, colourless
*V* = 686.48 (19) Å^3^	0.38 × 0.11 × 0.10 mm
*Z* = 2	

### Data collection

**Table:**

Bruker X8 APEX diffractometer	2698 reflections with *I* > 2σ(*I*)
Radiation source: fine-focus sealed tube	*R*_int_ = 0.031
Graphite monochromator	θ_max_ = 27.1°, θ_min_ = 2.7°
φ and ω scans	*h* = −7→7
11541 measured reflections	*k* = −15→15
3036 independent reflections	*l* = −11→12

### Refinement

**Table:**

Refinement on *F*^2^	Primary atom site location: structure-invariant direct methods
Least-squares matrix: full	Secondary atom site location: difference Fourier map
*R*[*F*^2^ > 2σ(*F*^2^)] = 0.045	Hydrogen site location: inferred from neighbouring sites
*wR*(*F*^2^) = 0.131	H atoms treated by a mixture of independent and constrained refinement
*S* = 1.06	*w* = 1/[σ^2^(*F*_o_^2^) + (0.0862*P*)^2^ + 0.0329*P*] where *P* = (*F*_o_^2^ + 2*F*_c_^2^)/3
3036 reflections	(Δ/σ)_max_ < 0.001
162 parameters	Δρ_max_ = 0.34 e Å^−^^3^
1 restraint	Δρ_min_ = −0.16 e Å^−^^3^

### Special details

**Table:**

Geometry. All s.u.'s (except the s.u. in the dihedral angle between two l.s. planes) are estimated using the full covariance matrix. The cell s.u.'s are taken into account individually in the estimation of s.u.'s in distances, angles and torsion angles; correlations between s.u.'s in cell parameters are only used when they are defined by crystal symmetry. An approximate (isotropic) treatment of cell s.u.'s is used for estimating s.u.'s involving l.s. planes.
Refinement. Refinement of *F*^2^ against ALL reflections. The weighted *R*-factor *wR* and goodness of fit *S* are based on *F*^2^, conventional *R*-factors *R* are based on *F*, with *F* set to zero for negative *F*^2^. The threshold expression of *F*^2^ > σ(*F*^2^) is used only for calculating *R*-factors(gt) *etc*. and is not relevant to the choice of reflections for refinement. *R*-factors based on *F*^2^ are statistically about twice as large as those based on *F*, and *R*- factors based on ALL data will be even larger.

### Fractional atomic coordinates and isotropic or equivalent isotropic displacement parameters (Å^2^)

**Table:**

	*x*	*y*	*z*	*U*_iso_*/*U*_eq_	
C2	0.0588 (3)	0.50820 (14)	0.02419 (18)	0.0314 (4)	
H2	0.1734	0.5611	0.0000	0.038*	
C4	0.2591 (3)	0.37310 (19)	−0.11371 (19)	0.0453 (5)	
H4A	0.3279	0.4210	−0.1786	0.054*	
H4B	0.2399	0.3005	−0.1567	0.054*	
C9	0.3831 (5)	0.3909 (3)	0.4739 (2)	0.0657 (7)	
H9A	0.5201	0.3606	0.5226	0.079*	
H9B	0.2942	0.4216	0.5439	0.079*	
H11	−0.153 (4)	0.608 (2)	−0.012 (3)	0.051 (8)*	
C1	0.1382 (3)	0.45493 (13)	0.16735 (17)	0.0289 (3)	
H1	0.0019	0.4272	0.2047	0.035*	
C3	0.0279 (3)	0.41938 (15)	−0.09006 (19)	0.0372 (4)	
H3	−0.0630	0.3592	−0.0565	0.045*	
C5	0.4180 (3)	0.36301 (17)	0.02114 (19)	0.0395 (4)	
H5A	0.5132	0.2982	0.0162	0.047*	
H5B	0.5143	0.4279	0.0323	0.047*	
C6	0.2821 (3)	0.35300 (14)	0.14433 (17)	0.0304 (3)	
C7	0.3361 (3)	0.27224 (16)	0.2586 (2)	0.0412 (4)	
C8	0.2506 (4)	0.2968 (2)	0.3985 (2)	0.0545 (6)	
H8A	0.2637	0.2308	0.4564	0.065*	
H8B	0.0925	0.3170	0.3840	0.065*	
C10	0.4472 (4)	0.4851 (2)	0.3774 (2)	0.0531 (6)	
H10A	0.5572	0.4567	0.3186	0.064*	
H10B	0.5204	0.5431	0.4355	0.064*	
C11	0.2506 (3)	0.53852 (15)	0.28073 (19)	0.0372 (4)	
C12	0.5456 (4)	0.2016 (2)	0.2643 (3)	0.0612 (6)	
H12A	0.5204	0.1334	0.3119	0.092*	
H12B	0.6694	0.2405	0.3140	0.092*	
H12C	0.5803	0.1859	0.1708	0.092*	
C13	−0.0927 (4)	0.4641 (2)	−0.2266 (2)	0.0575 (6)	
H13A	−0.0141	0.5281	−0.2558	0.086*	
H13B	−0.2443	0.4842	−0.2120	0.086*	
H13C	−0.0954	0.4080	−0.2977	0.086*	
C14	0.3584 (4)	0.64005 (19)	0.2140 (3)	0.0574 (6)	
H14A	0.2429	0.6824	0.1611	0.086*	
H14B	0.4661	0.6150	0.1529	0.086*	
H14C	0.4329	0.6856	0.2865	0.086*	
C15	0.0719 (4)	0.5824 (2)	0.3696 (3)	0.0636 (7)	
H15A	0.1416	0.6300	0.4416	0.095*	
H15B	0.0005	0.5214	0.4118	0.095*	
H15C	−0.0391	0.6235	0.3113	0.095*	
O1	−0.1435 (2)	0.56718 (13)	0.04280 (17)	0.0465 (4)	
O2	0.1605 (2)	0.24617 (10)	0.14695 (15)	0.0414 (3)	

### Atomic displacement parameters (Å^2^)

**Table:**

	*U*^11^	*U*^22^	*U*^33^	*U*^12^	*U*^13^	*U*^23^
C2	0.0338 (8)	0.0274 (8)	0.0338 (9)	−0.0005 (7)	0.0068 (6)	0.0023 (7)
C4	0.0540 (11)	0.0512 (12)	0.0321 (9)	0.0069 (9)	0.0119 (8)	−0.0066 (8)
C9	0.0770 (16)	0.0825 (18)	0.0347 (11)	0.0019 (14)	−0.0090 (10)	0.0007 (12)
C1	0.0297 (7)	0.0277 (7)	0.0300 (8)	−0.0025 (6)	0.0062 (6)	−0.0001 (6)
C3	0.0429 (10)	0.0379 (9)	0.0304 (9)	−0.0010 (7)	0.0020 (7)	−0.0009 (7)
C5	0.0379 (9)	0.0398 (9)	0.0419 (10)	0.0050 (8)	0.0100 (7)	−0.0051 (8)
C6	0.0321 (7)	0.0265 (7)	0.0317 (8)	−0.0036 (6)	−0.0003 (6)	−0.0027 (7)
C7	0.0462 (10)	0.0351 (10)	0.0403 (10)	−0.0033 (8)	−0.0060 (8)	0.0039 (8)
C8	0.0599 (13)	0.0623 (14)	0.0407 (11)	−0.0036 (11)	0.0022 (9)	0.0197 (10)
C10	0.0487 (11)	0.0572 (14)	0.0514 (13)	−0.0088 (9)	−0.0045 (9)	−0.0138 (10)
C11	0.0398 (9)	0.0374 (9)	0.0347 (9)	−0.0056 (7)	0.0052 (7)	−0.0098 (7)
C12	0.0595 (14)	0.0520 (13)	0.0681 (15)	0.0150 (11)	−0.0130 (11)	0.0080 (11)
C13	0.0698 (14)	0.0646 (14)	0.0352 (11)	0.0089 (12)	−0.0088 (9)	−0.0009 (10)
C14	0.0689 (14)	0.0398 (11)	0.0640 (14)	−0.0125 (11)	0.0086 (11)	−0.0075 (10)
C15	0.0596 (13)	0.0766 (17)	0.0561 (14)	0.0007 (12)	0.0141 (11)	−0.0262 (13)
O1	0.0504 (8)	0.0417 (8)	0.0490 (9)	0.0166 (6)	0.0121 (6)	0.0105 (7)
O2	0.0490 (8)	0.0270 (6)	0.0459 (7)	−0.0068 (5)	−0.0067 (6)	0.0019 (6)

### Geometric parameters (Å, º)

**Table:**

C2—O1	1.427 (2)	C7—C12	1.508 (3)
C2—C3	1.531 (2)	C7—C8	1.512 (3)
C2—C1	1.546 (2)	C8—H8A	0.9700
C2—H2	0.9800	C8—H8B	0.9700
C4—C3	1.526 (3)	C10—C11	1.558 (3)
C4—C5	1.529 (3)	C10—H10A	0.9700
C4—H4A	0.9700	C10—H10B	0.9700
C4—H4B	0.9700	C11—C15	1.525 (3)
C9—C8	1.523 (4)	C11—C14	1.552 (3)
C9—C10	1.539 (4)	C12—H12A	0.9600
C9—H9A	0.9700	C12—H12B	0.9600
C9—H9B	0.9700	C12—H12C	0.9600
C1—C6	1.528 (2)	C13—H13A	0.9600
C1—C11	1.582 (2)	C13—H13B	0.9600
C1—H1	0.9800	C13—H13C	0.9600
C3—C13	1.527 (3)	C14—H14A	0.9600
C3—H3	0.9800	C14—H14B	0.9600
C5—C6	1.503 (2)	C14—H14C	0.9600
C5—H5A	0.9700	C15—H15A	0.9600
C5—H5B	0.9700	C15—H15B	0.9600
C6—C7	1.478 (3)	C15—H15C	0.9600
C6—O2	1.481 (2)	O1—H11	0.72 (3)
C7—O2	1.455 (2)		
			
O1—C2—C3	113.36 (15)	C6—C7—C8	117.44 (17)
O1—C2—C1	106.45 (14)	C12—C7—C8	115.52 (19)
C3—C2—C1	110.40 (13)	C7—C8—C9	111.26 (19)
O1—C2—H2	108.8	C7—C8—H8A	109.4
C3—C2—H2	108.8	C9—C8—H8A	109.4
C1—C2—H2	108.8	C7—C8—H8B	109.4
C3—C4—C5	113.30 (14)	C9—C8—H8B	109.4
C3—C4—H4A	108.9	H8A—C8—H8B	108.0
C5—C4—H4A	108.9	C9—C10—C11	116.50 (19)
C3—C4—H4B	108.9	C9—C10—H10A	108.2
C5—C4—H4B	108.9	C11—C10—H10A	108.2
H4A—C4—H4B	107.7	C9—C10—H10B	108.2
C8—C9—C10	114.41 (18)	C11—C10—H10B	108.2
C8—C9—H9A	108.7	H10A—C10—H10B	107.3
C10—C9—H9A	108.7	C15—C11—C14	107.35 (18)
C8—C9—H9B	108.7	C15—C11—C10	109.69 (18)
C10—C9—H9B	108.7	C14—C11—C10	104.72 (17)
H9A—C9—H9B	107.6	C15—C11—C1	109.53 (15)
C6—C1—C2	109.41 (13)	C14—C11—C1	112.59 (15)
C6—C1—C11	114.05 (14)	C10—C11—C1	112.74 (15)
C2—C1—C11	114.57 (14)	C7—C12—H12A	109.5
C6—C1—H1	106.0	C7—C12—H12B	109.5
C2—C1—H1	106.0	H12A—C12—H12B	109.5
C11—C1—H1	106.0	C7—C12—H12C	109.5
C4—C3—C13	110.86 (16)	H12A—C12—H12C	109.5
C4—C3—C2	108.51 (15)	H12B—C12—H12C	109.5
C13—C3—C2	112.26 (17)	C3—C13—H13A	109.5
C4—C3—H3	108.4	C3—C13—H13B	109.5
C13—C3—H3	108.4	H13A—C13—H13B	109.5
C2—C3—H3	108.4	C3—C13—H13C	109.5
C6—C5—C4	109.51 (14)	H13A—C13—H13C	109.5
C6—C5—H5A	109.8	H13B—C13—H13C	109.5
C4—C5—H5A	109.8	C11—C14—H14A	109.5
C6—C5—H5B	109.8	C11—C14—H14B	109.5
C4—C5—H5B	109.8	H14A—C14—H14B	109.5
H5A—C5—H5B	108.2	C11—C14—H14C	109.5
C7—C6—O2	58.92 (11)	H14A—C14—H14C	109.5
C7—C6—C5	122.77 (16)	H14B—C14—H14C	109.5
O2—C6—C5	112.77 (14)	C11—C15—H15A	109.5
C7—C6—C1	120.48 (15)	C11—C15—H15B	109.5
O2—C6—C1	114.54 (12)	H15A—C15—H15B	109.5
C5—C6—C1	113.81 (14)	C11—C15—H15C	109.5
O2—C7—C6	60.64 (11)	H15A—C15—H15C	109.5
O2—C7—C12	115.87 (18)	H15B—C15—H15C	109.5
C6—C7—C12	121.17 (19)	C2—O1—H11	105 (2)
O2—C7—C8	114.42 (17)	C7—O2—C6	60.45 (11)

### Hydrogen-bond geometry (Å, º)

**Table:**

*D*—H···*A*	*D*—H	H···*A*	*D*···*A*	*D*—H···*A*
O1—H11···O2^i^	0.72 (3)	2.11 (3)	2.820 (2)	171 (3)

Symmetry code: (i) −*x*, *y*+1/2, −*z*.
